# The association between type 2 diabetes and dietary antioxidant index: a cross-sectional study in the Iranian population

**DOI:** 10.20945/2359-4292-2024-0170

**Published:** 2025-03-24

**Authors:** Zahra Roumi, Majid Kamali, Masoomeh Alsadat Mirshafaei, Saheb Abbas Torki, Bahareh Aminnezhad, Zahra Mahmoudi, Soheila Shekari, Ali Shamsi-Goushki, Kadijeh Abbasi Mobarakeh, Masoomeh Ataei Kachooei, Maryam Gholamalizadeh, Saeed Omidi, Parsa Bahmani, Saeid Doaei, Hamideh YazdiMoghaddam

**Affiliations:** 1 Department of Nutrition, Science and Research Branch, Islamic Azad University, Tehran, Iran; 2 Nutrition and Food Sciences Research Center, School of Nutrition and Food Sciences, Isfahan University of Medical Sciences, Isfahan; 3 Department of Sport Physiology, Tonekabon Branch, Islamic Azad University, Tonekabon, Iran; 4 Department of Nutrition, Faculty of Nutrition Sciences, Shiraz University of Medical Sciences, Shiraz, Iran; 5 Department of Nutrition, School of Medicine, Mashhad University of Medical Sciences, Mashhad, Iran; 6 Department of Community Nutrition, Nutrition and Food Security Research Center, School of Nutrition and Food Science, Isfahan University of Medical Sciences, Isfahan, Iran; 7 Faculty of Medicine, Shahrekord University of Medical Sciences, Shahrekord, Iran; 8 Cancer research center, Shahid Beheshti University of Medical Sciences, Tehran, Iran; 9 Department of Health Education and Promotion, Research Center of Health and Environment, School of Health, Guilan University of Medical Sciences, Rasht, Iran; 10 Shahid Beheshti University of Medical Sciences, Tehran, Iran; 11 Department of Community Nutrition, Faculty of Nutrition and Food Technology, National Nutrition and Food Technology Research Institute, Shahid Beheshti University of Medical Sciences, Tehran, Iran; 12 Reproductive Health Research Center, Department of Obstetrics and Gynecology, School of Medicine, Al-Zahra Hospital, Guilan University of Medical Sciences, Rasht, Iran; 13 Iranian Research Center on Healthy Aging, Operating Room Department, Faculty of Paramedics, Sabzevar University of Medical Sciences, Sabzevar, Iran

**Keywords:** Type 2 diabetes, diet, antioxidant, dietary antioxidant index

## Abstract

**Objective:**

This study aims to explore the association between dietary antioxidant index (DAI) and type 2 diabetes (T2D) in the Iranian population.

**Subjects and methods:**

The present cross- sectional study comprised 4,241 participants aged from 35 to 70. A food frequency questionnaire (FFQ) was used to assess dietary intake. The DAI score was determined using Wright’s method, which quantifies the antioxidant content of the diet. Logistic and linear regression analyses were used to determine the link between DAI and T2D after adjusting for confounding variables.

**Results:**

Negative associations were found between T2D with total score of DAI (OR = 0.67, CI95%: 0.55-0.81, P = 0.001) and DAI score of zinc (OR = 0.53, CI95%: 0.40-0.72, P = 0.001), manganese (OR = 0.77, CI95%: 0.68-0.88, P = 0.001), and selenium (OR = 0.88, CI95%: 0.78-0.98, P = 0.010) after adjustments for age, sex, BMI, education level, marital status, occupation, physical activity, and calorie intake.

**Conclusion:**

These results indicate the significance of an antioxidant-rich diet in preventing T2D and its complications. Nevertheless, additional investigation is required to validate these findings and explore the fundamental mechanisms of the association of T2D and dietary antioxidants.

## INTRODUCTION

Diabetes is defined by elevated levels of glucose in the blood, compromised tolerance to glucose, impaired secretion of insulin, and resistance to insulin (^1,2^). The prevalence of type 2 diabetes mellitus (T2D) increases with age and based on recent research, the prevalence of T2D was 29.8% in those over 65 (^3^) and 6.28% worldwide (1. According to the World Health Organization (WHO), non-communicable diseases (NCDs) accounted for 74% of global deaths in 2019, with diabetes causing 1.6 million deaths and ranking as the ninth most common cause of death worldwide.

Some studies predicted that nearly 592 million people will die from diabetes by 2035 (^4,5^).

Oxidative stress plays a crucial role in the development of type 2 diabetes by exacerbating both microvascular and macrovascular complications including diabetic retinopathy, nephropathy, and neuropathy, as well as cardiovascular issues like atherosclerosis and coronary heart disease (^6,7^). An increasing body of evidence from both experimental and clinical studies suggests that oxidative stress plays a significant role in the development of type 2 diabetes through various mechanisms. Specifically, an excessive production of free radicals combined with a weakening of antioxidant defense systems can lead to harmful impacts on cellular organelles and enzymes. Impaired antioxidant defense can cause prolonged lipid peroxidation, which is a process where free radicals steal electrons from the lipids in cell membranes, resulting in cell damage. Additionally, these oxidative processes are linked to the initiation of insulin resistance, a key factor in the development of type 2 diabetes (^8,9,10^). Additionally, compared to healthy individuals, individuals with type 2 diabetes had a significant decrease in overall antioxidant status and an increase in levels of oxidative stress markers; the levels of oxidative stress factors have been suggested as early predictors and indicators of complications associated with T2D (^11,12,13,14^).

Antioxidants act in preventing the generation of ex- cess free radicals, and hence avoiding oxidative damage to the cell (11. Previous studies indicated that increased consumption of common dietary antioxidants includ- ing vitamin E and vitamin C increased insulin sensitivity and reduced blood glucose and HbA1c levels (^15,16^). Another study reported that the composite dietary an- tioxidant index (CDAI) was negatively associated with diabetes and the relationship was independent of other traditional risk factors (17. Magnesium and zinc co- supplementation may be beneficial for patients with type 2 diabetes mellitus through antioxidant properties (18. However, the majority of studies examined the relation- ship between antioxidant-containing micronutrients or foods individually or in restricted dietary plans and found that a dietary regimen abundant in antioxidants may offer protection against the development of T2D (19. Considering that antioxidants can strengthen each other’s effect (^2^), total dietary antioxidants may have a different total effect compared with their individual ef- fects. As a result, the dietary antioxidant index (DAI) was designed to examine the diet’s complete antioxidant impact on oxidative stress state (^20,21,22^). There have been few investigations to establish the association be- tween DAI and T2D. Hence, we conducted the pres- ent cross-sectional study to investigate the association between the dietary antioxidant index and T2D in the Iranian population.

## SUBJECTS AND METHODS

### Study Population

This cross-sectional study was conducted in 2023 using Sabzevar Persian cohort data on 4,241 participants in the age range of 35 up to 70 years in Sabzevar, Iran. The inclusion criteria were to fill out a written informed consent and have a definite diagnosis of T2D. The exclusion criteria included participants who were unwilling to continue participating in the research, unable to provide the necessary information, had a daily energy consumption of less than 500 kcal or more than 5,000 kcal, or were using antioxidant supplements. Diabetes was diagnosed as FBS ≥ 6.99 mmol/L (^126^) mg/dL) according to the American Diabetes Association (ADA) 2020 criteria (23. Finally, 589 patients with T2D and 3,611 individuals without T2D were included.

### Data collection

Details concerning the socio-demographic and economic backgrounds of the participants were gathered through the Persian cohort questionnaire. To accurately measure physical characteristics, weight was assessed using the SECA 755 mechanical column scale, while height was measured with the SECA 204 mobile stadiometer. The body mass index (BMI) of each participant was calculated by weight (in kg) divided by the square of height in meters.

### Nutritional status

The dietary data used in the analysis was sourced from the Persian cohort’s food frequency questionnaire (FFQ), which has been validated and deemed reliable in previous studies (24. The frequency of food consumption over the past year was examined through a face-to-face interview. Household measures were taken into account for portion sizes and then were converted to grams. The food composition table (FCT) of the United States Department of Agriculture (USDA) was used to evaluate the amount of energy and nutrients. These data were then used to determine macro and micronutrient intake by the Nutritionist IV software (version 7.0; N-Squared Computing, Salem, OR, USA). To evaluate the antioxidant intake of the participants, Wright’s method was employed to calculate the Dietary Antioxidant Index (DAI) score. This index, which is considered a key independent variable in this study, quantifies the total antioxidant content of an individual’s diet. The process of calculating the DAI involved several detailed steps focusing on the intake of six specific nutrients known for their antioxidant properties: vitamins A, C, E, and the minerals manganese, selenium, and zinc. For each of these nutrients, the global average intake was first determined. This average was then subtracted from the actual intake amount reported by each participant for that particular nutrient. Following this subtraction, the difference obtained was divided by the standard deviation of that nutrient’s intake across the study population. This calculation yielded a standardized value for each nutrient, reflecting how much an individual’s intake deviated from the average. To derive the overall DAI score, these standardized values for all six nutrients were then summed.

### Statistical analysis

Shapiro-Wilk’s test was employed to assess the normality of the distributions for continuous variables. The demographic, social, and anthropometric characteristics of the participants were evaluated using the independent sample t-test for quantitative data and the chi-squared test for qualitative data. Logistic and linear regression methods were utilized to explore the association between the DAI and diabetes and fasting blood sugar (FBS), adjusting for potential confounders including age, sex, BMI, education level, marital status, occupation, physical activity, and calorie intake. All statistical analyses were conducted using SPSS version 22, and findings were deemed statistically significant at a probability level of P < 0.05.

## RESULTS

Table 1 presents the general characteristics of the participants. Patients with T2D had higher age, weight, BMI, right hand SBP, right hand DBP, WBC, FBS, TG, SGPT, and lower MET, height, HDLC, LDLC, cholesterol, occupation and education compared to the individuals without T2D (all P < 0.05).

**Table 1:** General characteristics of participants

	Patients with T2D (n = 589)	Individuals without T2D (n = 3611)	P
Age (year)	54.53 ± 7.75	48.36 ± 8.62	0.001
MET (kcal/kg/h)	37.25 ± 6.86	38.86 ± 7.91	0.001
Height (cm)	161.44 ± 9.37	162.33 ± 9.18	0.03
Weight (kg)	76.18 ± 13.49	73.77 ± 13.41	0.001
BMI (kg/m^2^)	29.24 ± 4.74	28.02 ± 4.70	0.001
Overweight/Obese	441 (74.9%)	2986 (82.7%)	0.001
Right hand SBP (mmHg)	121.61 ± 17.24	113.33 ± 16.71	0.001
Right hand DBP (mmHg)	75.33 ± 9.92	71.413 ± 10.53	0.001
WBC (k/µL)	6.72 ± 1.64	6.33 ± 1.57	0.001
RBC (m/µL)	4.89 ± 0.56	4.86 ± 0.54	0.23
FBS (mg/dL)	169.95 ± 69.28	96.87 ± 19.39	0.001
TG (mg/dL)	175.96 ± 114.74	141.61 ± 99.37	0.001
Cholesterol (mg/dL)	187.12 ± 46.19	192.45 ± 39.11	0.008
SGOT (IU/L)	19.61 ± 12.48	20.28 ± 8.21	0.21
SGPT (IU/L)	23.55 ± 15.96	21.88 ± 15.15	0.01
HDLC (mg/dL)	51.59 ± 10.55	52.52 ± 10.66	0.05
LDLC (mg/dL)	101.97 ± 36.54	111.68 ± 33.02	0.001
Has job (n, %)	186 (31.6)	1590 (44.0)	0.001
Male (n, %)	267 (45.3)	1604 (44.4)	0.68
Education (n, %)			
Diploma	172 (29.2)	817 (22.6)	0.001
Higher education	101 (17.1)	885 (24.5)	
Has hypertension	47.2%	19.4%	0.001
Has hyperlipidemia	39.1%	36%	0.079

SBP: systolic blood pressure; DBP: diastolic blood pressure; WBC: white blood cell; RBC: red blood cell; FBS: fasting blood sugar; TG: triglyceride; SGOT: serum glutamic oxaloacetic transaminase; SGPT: serum glutamic pyruvic transaminase; HDLC: high-density lipoprotein; LDLC: low-density lipoprotein cholesterol.

Dietary intake among patients with type 2 diabetes and individuals without T2D is presented in Table 2. The patients had lower intake of protein, fat, carbohydrate, energy, zinc, and selenium compared to the individuals without T2D (all P < 0.05). As shown in Table 3, the patients with T2D had lower DAI scores for zinc and selenium compared to the individuals without T2D (Both P < 0.01).

**Table 2:** Dietary intake among patients with diabetes and individuals without T2D

	Patients with T2D (n = 589)	Individuals without T2D (n = 3,611)	P
Calorie intake (kcal/kg/d)	31.81 ± 11.02	34.78 ± 11.18	0.001
Protein intake (g/kg/d)	1.02 ± 0.36	1.09 ± 0.36	0.001
Fat intake (g/kg/d)	0.82 ± 0.35	0.9 ± 0.36	0.001
Carbohydrate intake (g/kg/d)	5.25 ± 1.93	5.75 ± 1.96	0.001
Calorie (kcal/d)	2381.7 ± 802.17	2516.77 ± 780.71	0.001
Protein (g/d)	76.60 ± 25.97	78.99 ± 26.06	0.04
Fat (g/d)	61.47 ± 26.28	64.99 ± 25.13	0.003
Carbohydrate (g/d)	393.16 ± 140.01	415.90 ± 137.51	0.001
Zinc (mg/d)	9.75 ± 3.39	10.13 ± 3.38	0.01
Manganese (mg/d)	5.73 ± 2.01	5.64 ± 1.92	0.35
Selenium (mg/d)	50.95 ± 29.43	55.79 ± 28.62	0.001
Vitamin A (IU/d)	8984.13 ± 5957.11	8651.44 ± 5433.79	0.21
Vitamin E(mg/d)	7.008 ± 3.26	7.21 ± 3.25	0.15
Vitamin C (mg/d)	147.15 ± 94.63	140.91 ± 78.96	0.13

**Table 3:** The levels of dietary antioxidant index among patients with T2D and individuals without T2D

	Patients with T2D (n = 589)	Individuals without T2D (n = 3,611)	P
Total DAI	-0.029	-0.002	0.43
Vitamin C DAI	0.064 ± 1.16	-0.013 ± 0.97	0.13
Vitamin A DAI	0.04 ± 1.06	-0.01 ± 0.97	0.21
Vitamin E DAI	-0.05 ± 1.01	0.005 ± 1.01	0.15
Zinc DAI	-0.1003 ± 0.99	0.0104 ± 0.99	0.01
Selenium DAI	-0.14 ± 1.02	0.02 ± 0.99	0.001
Manganese DAI	0.01 ± 1.06	-0.02 ± 1.01	0.35
Total DAI tertiles			
First tertile (DAI < -0.386)	-0.77 ± 0.33	-0.73 ± 0.26	0.176
Second tertile (-0.386-0.172)	-0.10 ± 0.15	-0.12 ± 0.16	0.172
Third tertile DAI > 0.172)	0.84 ± 0.69	0.84 ± 0.65	0.89

Regarding the association between T2D and dietary antioxidant status, negative associations were found between T2D with total score of DAI (OR = 0.67, CI95%: 0.55-0.81, P = 0.001) and DAI score of zinc (OR = 0.53, CI95%: 0.40-0.72, P = 0.001), manganese (OR = 0.77, CI95%: 0.68-0.88, P = 0.001), and sele- nium (OR = 0.88, CI95%: 0.78-0.98, P = 0.010) after adjustments for education level, marital status, occu- pation, physical activity, and calorie intake, (Model 2). The association between T2D and DAI scores for vita- min C, vitamin A, and vitamin E was not found to be statistically significant (Table 4). Linear regression of the association between DAI and FBS found an inverse association between the level of FBS and the DAI score of vitamin E (β = -0.083, P=0.031), zinc (β = -0.027, P = 0.037), selenium (β = -2.072, P = 0.038), and mag- nesium (β = -0.050, P = 0.018).

**Table 4:** The association of type 2 diabetes and dietary antioxidant index scores

	Type 2 diabetes (as a categorical variable)^α^		Fasting blood sugar (as a continuous variable)^β^	
	OR (^95^)% CI)	P*	β	P*
Total DAI score	0.67 (0.55-0.81)	0.001	1.478	0.139
Vitamin C	0.90 (0.79-1.03)	0.13	0.034	0.139
Vitamin A	0.98 (0.86-1.12)	0.82	-.007	0.849
Vitamin E	0.96 (0.82-1.11)	0.59	-.083	0.031
Zinc	0.53 (0.40-0.72)	0.001	-.027	0.037
Selenium	0.88 (0.78-0.98)	0.01	-2.072	.038
Magnesium	0.77 (0.68-0.88)	0.001	-0.050	0.018

α Logistic regression. ^β^ Linear regression. * Adjusted for age, sex, BMI, education level, marital status, occupation, physical activity, and calorie intake.

## DISCUSSION

According to our information, this is the first cross- sectional study to investigate the association between DAI and T2D in the Iranian population. In the present study, a significant association was observed between T2D and the total score of DAI after adjusting for confounding factors. Specifically, DAI score of zinc, selenium and manganese also had a significant negative association with T2D after adjustment of confounding factors. The results of the present study are consistent with the findings of a number of studies that have been conducted in recent years and have shown that the consumption of antioxidants may have an inverse relationship with the risk of developing T2D. However, some studies used the Dietary Total Antioxidant Capacity (DTAC) index for the assessment of this association and some other research investigated the effect of receiving each substance with antioxidant properties on diabetes separately (25. Previous studies reported the link between vitamin C and E intake through diet increased insulin sensitivity and improved HbA1c levels (^15,16^). The DTAC of a Polish population was shown to have a positive association with the individual dietary antioxidants that were consumed, which included polyphenols, antioxidant vitamins, and minerals and decreased DTAC was reported to be an additional risk factor for developing T2D (26. According to the findings of a meta-analysis, the consumption of antioxidants was primarily associated with a 13% decrease in the risk of developing diabetes. This reduction was most closely associated with the consumption of vitamin E and carotenoids via the diet (27. In addition, some studies found that consuming fruits and vegetables that contain favorable antioxidant properties (^28^) and also the intake of magnesium (^29^), zinc (^30^), and selenium (^31^) can regulate the inflammatory cascade and oxidation, which highlights the effect of a healthy food pattern on diabetes. Our study also concluded that DAI, which comprises manganese, selenium, vitamins A, C, and E, and zinc, had an adverse association with type 2 diabetes. However, this link was not significant separately for vitamin A and vitamin C. In this regard, it possible that some antioxidants may have synergistic effects (32.

The association between the consumption of antioxidants in the diet and the risk of developing type 2 diabetes may be attributed to the impact of oxidative stress on T2D. Dietary antioxidants exert their effects via two mechanisms: firstly, by inhibiting the generation of excessive free radicals and oxidative damages in cells; and secondly, by mitigating additional destruction and minimizing the progression of oxidative stress once damage has occurred (33. A considerable reduction in C-reactive protein (CRP) levels and a substantial elevation in total antioxidant capacity (TAC) and total nitrite were observed in patients diagnosed with coronary heart disease and type 2 diabetes mellitus over the course of 12 weeks following supplementation with combined magnesium and zinc (34.

Despite the strengths of this article, this research had some limitations. First, dietary assessment may be influenced by measurement error and low accuracy. Another limitation was the lack of knowledge of cooking methods by the participants, which can affect the antioxidant content of foods. Third, the drugs used by the participants in the study were not assessed and adjusted in the analyses. Fourth, the association between the intake of antioxidants and some indicators related to diabetes, such as HOMA-IR was not investigated. Finally, this was an observational study and the cause and effect relationship between diet and outcomes can only be established through randomized clinical trials. Future longitudinal studies would be needed to confirm the association between T2D and DAI score.

In conclusion, this study showed that DAI may have a significant association with the low risk of T2D after adjusting for confounding factors. Therefore, this index along may be useful in making effective dietary recommendations to prevent and control T2D. Additional research is necessary to validate this finding and uncover the underlying mechanism by which dietary antioxidants affect T2D. Also, randomized clinical trials are needed before anti-oxidant rich diets can be recommended.
